# Apathy symptoms induced by low‐dose venlafaxine: Two cases

**DOI:** 10.1002/npr2.12104

**Published:** 2020-04-08

**Authors:** Shinji Sato, Noriko Sodeyama, Asaki Matsuzaki, Yuki Shiratori

**Affiliations:** ^1^ Department of Psychiatry Ibaraki Clinical Educational and Training Center Faculty of Medicine University of Tsukuba Tsukuba Japan; ^2^ Department of Psychiatry Ibaraki Prefectural Hospital Kasama City Japan; ^3^ Department of Psychiatry Division of Clinical Medicine Faculty of Medicine University of Tsukuba Tsukuba Japan

**Keywords:** apathy, norepinephrine, serotonin, SSRIs induced apathy, venlafaxine

## Abstract

Recent guidelines regarding pharmacological interventions for major depressive disorder (MDD) recommend first using serotonin (5HT) selective reuptake inhibitors (SSRIs) or 5HT and norepinephrine (NE) reuptake inhibitors (SNRIs). Although SSRIs and SNRIs are effective and well‐tolerated, apathy occurs as an adverse effect in some SSRIs‐treated patients. Because apathy would be associated with the 5HT pathway, if a patient exhibits apathy symptoms under SSRIs treatment, a clinical strategy has been to change the SSRIs to treatment with an SNRIs. Here, I report two cases in which low‐dose venlafaxine, an SNRIs, induced apathy symptoms.

Recent guidelines regarding pharmacological interventions for major depressive disorder (MDD) recommend first using serotonin (5HT) selective reuptake inhibitors (SSRIs) or serotonin and norepinephrine reuptake inhibitors (SNRIs).^1^, ^2^ Although SSRIs are effective and well‐tolerated, apathy occurs as an adverse effect in some SSRIs‐treated patients.^3^ Because apathy might be associated with the 5HT pathway through a relatively hypodopaminergic and/ or hyponorepinephrine state,^4^ if a patient exhibits apathy symptom under treatment with SSRIs, the clinical strategy has been to reduce the dosage of SSRIs or to change the SSRIs to treatment with SNRIs.^3^, ^5^ Here, I report two cases in which low‐dose venlafaxine, an SNRIs, induced apathy symptoms. All the patients were given informed consent, and their anonymity was preserved.

Mr A, a 47‐year‐old male, was referred as an outpatient with MDD according to the DSM‐5 criteria, and his MADRS was 24 point. He was first treated with mirtazapine (up to 45 mg) but showed slight losses of initiative and motivation. The mirtazapine dose was thus tapered gradually, and venlafaxine was added at dosage of 37.5 mg/d. One month after the mirtazapine was discontinued and he began taking 75 mg venlafaxine daily, he reported feeling a slight loss of motivation and not experiencing emotions without a worsening of his depressive mood and anxiety. He also described not feeling any suffering or considering anything deeply. At that time, his total MADRS decreased to 12. Though these phenomena occurred, he decided to continue taking venlafaxine because his depressive mood and anxiety had not worsened. Two weeks after that, these emotion‐related phenomena gradually improved, and the venlafaxine dosage was increased to 150 mg upon his request. Two weeks later the patient reported that his depressive symptoms were improved, and his MADRS was 6 point.

Mr B, a 55‐year‐old male referred with MDD according to the DSM‐5 criteria, was administered venlafaxine. His MADRS score was firstly 22. However, after taking 37.5 mg of venlafaxine for 2 weeks, his losses of motivation slightly worsened, and both his sadness and pleasure were blunted with contrasting slight improvements of his depressive mood and anxiety symptoms. He insisted that he keep taking venlafaxine because he was showing improvements in his depressive mood and anxiety. And his score of MADRS reduced to 10 point. When the venlafaxine dosage was increased to 75 mg, he reported a gradual return of both motivation and emotions. At 150 mg venlafaxine, his depressive symptoms were improved.

Although venlafaxine is an SNRIs antidepressant, it has moderate affinity for 5HT transporters and low affinity for norepinephrine (NE) transporters.^6^ In the human brain, the 5HT transporter occupancy of venlafaxine at 75 mg/d already reaches 80%, and at higher dosages it reaches a plateau of occupancy. On the other hand, the NE transporter occupancy of venlafaxine varies in a dose‐dependent manner, and there is a significant difference between higher doses (150 mg/d or more) and lower doses (75 mg/d or less) of venlafaxine.^7^ In the cases described above, it is possible that, with the administration of a low venlafaxine dose, because of the stronger reuptake inhibition effects of 5HT compared to NE, mild apathy‐like symptoms might have occurred as an adverse effect of 5HT and a slight effect of NE. In case 1, it could be considered that the discontinuation of mirtazapine induced a worsening of his disturbance of motivation and flattened his emotion; however, his depressive mood and anxiety did not change. In case 2, with a low dosage of venlafaxine, the patient felt his emotions slightly blunted in contrast to the improvements of his other depressive symptoms. Up to the maximum dose of venlafaxine, all of his symptoms were reduced. Thus, the temporary symptom which occurred with the low dosage of venlafaxine would be considered apathy induced by unbalanced concentrations of 5HT and NE.

When more venlafaxine is added, the mild apathy symptom induced by 5HT might be reduced. If patients exhibit slight apathy symptoms such as blunted emotions or loss of motivation and also show improvement of their other depressive symptoms while under venlafaxine treatment, clinicians should consider two options: to continue the venlafaxine administration with a dose adjustment so as not to waste time by changing medications, or to change to another SSRIs or SNRIs to avoiding worsening depressive symptoms.^5^, ^7^ If a patient who takes a low dose of venlafaxine shows apathy without the worsening of any other depressive symptoms, it would be useful to keep the possibility of increasing the dosage of venlafaxine in mind.

## CONFLICT OF INTEREST

The author declares no conflict of interest.

## AUTHOR CONTRIBUTIONS

SS treated two cases and wrote the initial draft of the manuscript. NS, AM, and YS contributed to interpretation and assisted in the preparation of the manuscript. All authors have contributed to cases interpretation and critically reviewed the manuscript. All authors approved the final version of the manuscript. All authors agreed to be accountable for all aspects of the work in ensuring that questions related to the accuracy or integrity of any part of the work are appropriately investigated and resolved.

## Data Availability

The data that supports the findings of this study are included in this article.
